# Toxicity Evaluation and Controlled-Release of Curcumin-Loaded Amphiphilic Poly-N-vinylpyrrolidone Nanoparticles: In Vitro and In Vivo Models

**DOI:** 10.3390/pharmaceutics16010008

**Published:** 2023-12-19

**Authors:** Anna L. Luss, Dmitry V. Bagrov, Anne V. Yagolovich, Ekaterina V. Kukovyakina, Irina I. Khan, Vadim S. Pokrovsky, Maria V. Shestovskaya, Marine E. Gasparian, Dmitry A. Dolgikh, Andrey N. Kuskov

**Affiliations:** 1 Department of Technology of Chemical, Pharmaceutical and Cosmetic Substances, D. Mendeleev University of Chemical Technology of Russia, 125047 Moscow, Russia; kev0700@yandex.ru (E.V.K.); marieshestovskaya@gmail.com (M.V.S.); marine_gasparian@yahoo.com (M.E.G.); kuskov.a.n@muctr.ru (A.N.K.); 2 Faculty of Biology, Lomonosov Moscow State University, 119234 Moscow, Russia; bagrov@mail.bio.msu.ru (D.V.B.); anneyagolovich@gmail.com (A.V.Y.); dolgikh@nmr.ru (D.A.D.); 3 N.N. Blokhin National Medical Research Center of Oncology, Ministry of Health of Russia, 115478 Moscow, Russiavadimpokrovsky@yandex.ru (V.S.P.); 4 Department of Biochemistry, People’s Friendship University of Russia (RUDN University), 117198 Moscow, Russia; 5 Shemyakin-Ovchinnikov Institute of Bioorganic Chemistry of the Russian Academy of Sciences, 117997 Moscow, Russia

**Keywords:** curcumin, poly(N-vinylpyrrolidone), nanoparticles, glioblastoma, toxicity

## Abstract

Curcumin attracts huge attention because of its biological properties: it is antiproliferative, antioxidant, anti-inflammatory, immunomodulatory and so on. However, its usage has been limited by poor water solubility and low bioavailability. Herein, to solve these problems, we developed curcumin-loaded nanoparticles based on end-capped amphiphilic poly(N-vinylpyrrolidone). Nanoparticles were obtained using the solvent evaporation method and were characterized by dynamic and electrophoretic light scattering, transmission electron (TEM) and atomic force (AFM) microscopy. The average particle size was 200 nm, and the ζ-potential was −4 mV. Curcumin-release studies showed that nanoparticles are stable in aqueous solutions. An in vitro release study showed prolonged action in gastric, intestinal and colonic fluids, consistently, and in PBS. In vitro studies on epidermoid carcinoma and human embryonic kidney cells showed that the cells absorbed more curcumin in nanoparticles compared to free curcumin. Nanoparticles are safe for healthy cells and show high cytotoxicity for glioblastoma cells in cytotoxicity studies in vitro. The median lethal dose was determined in an acute toxicity assay on zebrafish and was 23 μM. Overall, the curcumin-loaded nanoparticles seem promising for cancer treatment.

## 1. Introduction

In recent years, micelles have been studied as one of the most promising strategies for site-specific drug delivery. One of the most well-known polymers for creating micellar delivery systems is polyethylene glycol (PEG) [1,2,3]. Despite the well-studied chemistry of PEG, there is currently an active search for alternative polymers/copolymers for the delivery of physiologically active substances with similar properties. Polylactide [4,5], poly(lactide-co-glycolide) [6,7,8], poly(ε-caprolactone) [9,10] and others are also used as carriers.

In the last decade, interest has arisen in carriers based on poly(N-vinylpyrrolidone) (PVP) [11,12]. Like PEG, PVP has a very long history of medical use [13,14]. From a pharmaceutical point of view, it has attracted significant interest due to certain physicochemical properties: solubility in almost all solvents, affinity for hydrophobic and hydrophilic surfaces, ability to form complexes, superior bioavailability and biocompatibility [13]. PVP is widely used in medicine as an antiseptic in combination with iodine and as a component of solutions for contact lenses [15,16]. Moreover, it is included in the FDA’s database of inactive ingredients for use in oral, topical and injectable pharmaceutical products [17].

Amphiphilic PVP derivatives with hydrophobic terminal groups have been studied over the past decades as intravenous drug delivery systems [18,19,20]. They self-organize in aqueous solutions to form micellar structures and demonstrate excellent hemo- and biocompatibility. Compared to standard nanoparticles (PLGA, PLA, etc.), such aggregates have a hydrophilic outer shell and inner hydrophobic core, which allows the encapsulation of hydrophobic substances with high efficiency without any stabilizers. Parameters such as the copolymer molecular weight, morphology and size of the micelles are easily controlled during the synthesis process. They can capture various therapeutic agents, such as non-steroidal anti-inflammatory drugs [19,20,21], antitumor drugs [22], antifungal antibiotics [23], cytokines [24,25] and plasmid DNA [26] in micelles. Depending on the average hydrodynamic radius, nanoparticles can be absorbed by cell cultures through various mechanisms, including endocytosis and membrane fusion, which allows selective delivery of the active substance to both the cell’s endosomes and its nucleus [27]. In addition, PVP nanoparticles can be used as effective modifiers of liposomal membranes, increasing their stability [23]. 

Curcumin is a natural polyphenol found in turmeric. Its clinical potential is determined by anti-inflammatory [28], antimicrobial [29], anticarcinogenic [30,31], hypoglycemic [32,33] and immunomodulating [34] and other properties. Curcumin has a very promising property, namely increased uptake by tumor cells in vitro. Moreover, its pro-apoptotic effect increases with increasing intracellular concentration, which may be promising in the development of drugs against malignant neoplasms, including glioblastoma and colorectal cancer [35,36,37,38,39]. Curcumin has also been shown to be effective not only as an anticancer drug but also in the fields of oral hygiene, periodontal therapy, gastrointestinal diseases, ophthalmic drugs and wound healing [37]. Despite its wide range of biological activities, curcumin has low bioavailability due to its poor solubility in aqueous media and low stability in the presence of air and UV radiation [40,41]. These limitations represent a major problem to the study and application of curcumin in biomedicine. A solution to this problem could be the effective encapsulation of curcumin into nanoparticles [42]. 

Herein, we report the preparation of curcumin-loaded nanoparticles based on amphiphilic PVP end-capped with a tiooctadecyl group. PVP with a molecular weight of 6 kDa was selected based on previous studies as the most suitable candidate to achieve maximum encapsulation of curcumin by its lipophilic–lyophobic balance. Nanoparticles were prepared by the solvent evaporation method. The anticancer activity of curcumin-loaded PVP was estimated in glioblastoma cell lines. In addition, in vitro release dynamics were measured in various environments, and the acute toxicity of zebrafish in vivo was noted.

## 2. Materials and Methods

### 2.1. Materials

N-vinylpyrrolidone and octadecyl mercaptan (ODM) were obtained from Sigma-Aldrich (St. Louis, MO, USA). Azobiisobutyronitrile (AIBN) was obtained from Chemical Line (St. Petersburg, Russia). 1,4-dioxane was obtained from LenReaktiv (St. Petersburg, Russia). Curcumin was obtained from MT BIO-TECH (Changsha, China). All chemicals were used without further purification unless otherwise stated. Phosphate-saline buffer (PBS) were obtained from Servicebio (Wuhan, China). All solvents and components of buffer solutions were used as received.

### 2.2. Synthesis and Physicochemical Characteristics of Nanoparticles Based on Amphiphilic Poly(N-vinylpyrrolidone)

#### 2.2.1. Synthesis of Amphiphilic Poly(N-vinylpyrrolidone)

The synthesis of amphiphilic poly(N-vinylpyrrolidone) with a molecular weight of 6 kDa was carried out in accordance with [18,27,43]. Briefly, 20 mL of N-vinylpyrrolidone, AIBN 1 wt% (0.7 mol%) and ODM 0,25 wt% (1 mol%) were dissolved in 40 mL of 1,4-dioxane. The reaction was carried out at 70°C for 3 h. The resulting solution was dialyzed against water, frozen and freeze-dried. Molecular weight was determined by reverse iodometric titration according to the method described by the authors in [44] and it was 6 kDa. The absence of solvent residues was determined by thermogravimetric analysis.

#### 2.2.2. Synthesis of Curcumin-Loaded Nanoparticles Based on Amphiphilic Poly(N-vinylpyrrolidone) 

A 90 mg sample of amphiphilic poly(N-vinylpyrrolidone) was dissolved in 30 mL of water (3 mg/mL), and 4 mL of curcumin solution in acetone (2.5 mg/mL) was added to the resulting solution. Then the resulting mixture was dispersed on an ultrasonic homogenizer Bandelin SONOPULS HD 4400 (Berlin, Germany) in the 1-s-on/1-s-off mode with an amplitude of 25% for 20 min. Then, the solvent was distilled off using a Heidolph Hei-VAP Ultimate rotary evaporator (Schwabach, Germany), and the resulting suspension of nanoparticles (PVP-Cur NPs) was centrifuged at 4000 rpm. Supernatant was frozen and freeze-dried.

#### 2.2.3. Determination of Particle Size and Surface Charge

The ζ-potential and hydrodynamic diameter of the nanoparticles were determined by dynamic light scattering (DLS) and electrophoretic light scattering (ELS) using a Malvern Zetasizer Nano Z&S (Worcestershire, UK). Measurements were carried out at 25 °C and in distilled water three times for each sample.

#### 2.2.4. Transmission Electron Microscopy and Atomic Force Microscopy

The PVP-Cur NPs were visualized using transmission electron microscopy (TEM) and atomic force microscopy (AFM). For both imaging procedures, the NPs were deposited onto carbon–formvar TEM grids (mesh 200, Electron Microscopy Science, Hatfield, PA, USA). The grids were treated using a glow discharge device K100X (Emitech, currently Quorum Technologies, Laughton, UK) at 30 mA for 30 s. For the TEM imaging, the sample was diluted in water to c = 0.1 mg/mL, deposited onto the grids for approximately 1 min and then the grids were stained by 1% uranyl acetate and dried. Images was carried out using a JEM-1400 electron microscope (JEOL, Tokyo, Japan) operating at 120 kV, equipped with Rio-9 camera (Gatan Inc., Pleasanton, CA, USA).

For the AFM imaging, the sample was diluted in water to c = 0.25 mg/mL, deposited onto the grids for approximately 1 min and then the grids were washed with water and dried. The images were acquired in semicontact mode, using a Solver PRO-M microscope (NT-MDT, Zelenograd, Russia). The scanning rate was 1–1.7 Hz, and the NSG10 cantilevers (Tips-Nano, Moscow, Russia) were used (typical curvature radius 6 nm, typical force constant k = 11.8 N/m).

Image processing was carried out using Fiji [45] and FemtoScan Online [46] for the TEM images and AFM frames, respectively.

#### 2.2.5. Evaluation of Curcumin Encapsulation in the PVP-Cur NPs 

The curcumin encapsulation efficiency was calculated by ratio of total curcumin content in PVP-Cur NPs to total drug amount according to [47]. The percent of loaded drug was calculated from the total amount of drug extracted from the PVP-Cur NPs to the known weight of the nanoparticles. To extract curcumin, lyophilized PVP-Cur NPs were dissolved in acetonitrile (5 mg in 5 mL). The samples were stirred at 500 rpm for 2 h for full curcumin extraction. Then the samples were centrifuged at 10,000 rpm and supernatants were collected. Suspension (20 μL) was dissolved in ethanol (1 mL) and used for further estimations. The curcumin concentrations were measured spectrophotometrically at 425 nm. 

Curcumin loading efficiency (*LE*) was calculated using the following equation:

$$LE\left(\%\right)=\frac{Total\;curcumin\;content\;in\;NPs}{Total\;cucrcumin\;amount}\times 100\%$$

Curcumin loading capacity (*LC*) in the preparations was calculated using the following equation:

$$LC\left(\%\right)=\frac{Curcumin\;content}{Weight\;of\;nanoparticles}\times 100\%$$

### 2.3. Curcumin In Vitro Release Study 

#### 2.3.1. Curcumin Release in PBS Solution 

The curcumin in vitro release profile from the PVP-Cur NPs was obtained using dialysis technique as described [48]. PVP-Cur NPs were resuspended in 5 mL of PBS (pH 7.4) and dialyzed using Thermo Fisher Scientific (Waltham, MA, USA) tubes with a molecular weight cut-off (MWCO) of 1000 Da. During dialysis, the tubes were placed in a glass with 150 mL of PBS and incubated at 37 °C under constant shaking. The amount of curcumin released from PVP-Cur NPs was estimated by taking out 1.0 mL of buffer media at predetermined time intervals (0.5, 1, 2, 3, 4, 6, 8, 10, 12, 16, 20, 24, 36, 48 h). The content of the released curcumin in selected samples was determined by spectrophotometry at 425 nm. Free curcumin dissolved in methanol/water mixture at the same amount as in PVP-Cur NPs was used as a control. All samples’ measurements were run in triplicate. The standard curcumin calibration curve was obtained and used as a reference in the experiments.

#### 2.3.2. In Vitro Curcumin Release in Simulated Gastric, Intestinal and Colonic Digestion

In vitro curcumin release from PVP-Cur NPs was estimated as described by the authors in [49] using simulated gastric fluid (SGF, pH = 1.2) without enzymes (0.2% Polysorbate 80) for 2 h, simulated intestinal fluid (SIF, pH = 6.8) without enzymes (0.2% Polysorbate 80) for 6 h and simulated colonic fluid (SCF, pH = 7.4) (0.2% Polysorbate 80) with β-Galactosidase (0.13 units/mL) for 24 h at constant shaking and at 37 °C. Release medium was centrifuged, and the absorbance was measured spectrophotometrically at 425 nm to determine the amount of the released curcumin. All samples’ measurements were run in triplicate.

#### 2.3.3. Stability Studies 

Stability characteristics of PVP-Cur NPs were evaluated according to the International Council for Harmonisation of Technical Requirements for Pharmaceuticals for Human Use (ICH) guidelines (2003) code Q1A(R2) (stability testing of new drug substances and products). Nanoparticle samples were stored preserved from light in closed impenetrable tubes. For the accelerated condition experiment, samples were taken at 0, 1, 2, 4 and 6 months (40 ± 2 °C, 75% relative humidity ± 5%). The amount of curcumin released from the samples was measured using a UV-Vis spectrophotometer (UNICO 2804, United Products & Instruments Inc., Dayton, NJ, USA) at 425 nm.

#### 2.3.4. Water Resuspendability Study

Freeze-dried PVP-Cur NPs samples (10 mg) were dispersed in 10 mL of distilled water) and stirred for 5 min to test the homogeneity of the obtained suspensions using Zetasizer Nano Z&S (Malvern Instruments, Worcestershire, UK).

### 2.4. In Vitro and In Vivo Assays 

#### 2.4.1. Cell Lines

A431 epidermoid carcinoma cells (ATCC no.CRL-1555™) were cultured in DMEM with 4 mM L-glutamine supplemented with 10% FBS. Human embryonic kidney cells HEK 293 (ATCC no. CRL-1573™) were cultured in in Eagle’s Minimum Essential Medium, and human glioblastoma cell lines T98G (ATCC no. CRL-1690™) and U87 (ATCC no. HTB-14™) were cultivated in Dulbecco’s Modified Eagle Medium (DMEM) with 2 mM glutamine supplemented with 10% FBS. Cells were cultured at 37 °C in 5% CO_2_ in a humidified atmosphere. All cells were passaged with 0.25% *v*/*w* trypsin—0.53 mM EDTA at 80% confluence. 

Mouse embryonic fibroblasts NIH/3T3 cells (ATCC no. CRL-1658 ™) were chosen as a control healthy cell line. NIH/3T3 were cultured in DMEM supplemented with 10% FBS, l-glutamine (1 mM) and penicillin–streptomycin (100 U/mL). Cells were incubated at 37 °C in a humidified atmosphere containing 5% CO_2_ and split every 3 days using EDTA solution.

#### 2.4.2. Cytotoxicity Assay

The three cell lines (NIH/3T3, T98G and U87) were used in the cytotoxicity assay.

Viability of cells was estimated by measuring activity of mitochondrial NAD(P)H-dependent cellular oxidoreductase enzymes which could reduce a yellow tetrazolium salt ((3-(4,5-dimethyl-2-thiazolyl)-2,5-diphenyl-2-H-tetrazolium bromide or MTT) to a violet formazan dye by metabolically active cells.

Cells were seeded in a 96-well plate in the amount of 8 × 10^3^ cells per well. After overnight incubation, the studied substances (PVP, curcumin, PVP-Cur NPs) were added to cells in various concentrations in 100 µL of culture medium. After 24 h of drug exposition, cells were treated with 20 µL of MTT solution (MTT Cell Viability Assay Kit, Servicebio, Wuhan, China). In parallel, MTT was added to the wells with DMEM with NPs without cells that were used further for optical compensation. Incubation with MTT lasted for 4 h, then the medium was aspirated from the wells and formazan crystals were dissolved in dimethyl sulfoxide (DMSO). The compensation for DMSO absorbance was also performed. Optical density was measured at 570 nm on a Tecan Infinite^®^ M Nano + spectrophotometer (Männedorf, Switzerland). Absorption values were converted to percentages relative to absorption values of intact cells.

#### 2.4.3. Cell Uptake Assay

Cell uptake assay was performed on A431 and HEK 293 cell lines according to our previous work [27]. Briefly, the cells were seeded in a 96-well polystyrene plate at a density of 5 × 10^3^ cells per well. After 24 h, the cells were washed with PBS and then preincubated for 15 min with Hoechst 33258 dissolved in the cell medium at a ratio of 1:1000 and then washed with the medium. After that, 150 µL of DMEM supplemented with either free curcumin or with the PVP-Cur NPs was added to the wells. To analyze the distribution of curcumin, the cells were washed three times with PBS and then visualized using a Zeiss Axio Observer.Z1 inverted microscope (Oberkochen, Germany).

#### 2.4.4. Fish Embryo Acute Toxicity Test (FET)

Studies were conducted on zebrafish (*Danio rerio*) in accordance with OECD guideline Protocol 236 “Fish Embryo Acute Toxicity (FET) Test” (OECD/OCDE, 2013). Morphological effects were assessed according to [50].

Adult wild-type zebrafish were kept in aquariums with an aeration and recirculation system at a temperature of 28 °C, pH 6.5–7.5, with a photoperiod cycle of 14:10 h (light: dark). The fish were fed twice daily according to conventional recommendations (using zebrafish food).

Freshly laid eggs after fertilization (less 1 h post-fertilization (hpf)) were collected and placed in *Danio rerio* E3 embryo water (5 mM NaCl, 0.33 mM CaCl_2_, 0.33 mM MgSO_4_ · 7H_2_O and 0.17 mM KCl and 0.1% methylene blue). Unfertilized eggs and embryos that had significant developmental defects 24 h after fertilization were detected under a Nexcope NSZ-810 microscope (Ningbo, China) and removed from the experiment. Experimental embryos were mechanically dechorionized with tweezers and placed in 24-well plates (2 embryos per well, total 0.5–1.5 mL of solution per well). PVP-Cur NPs nanoparticles were added in quantities such that final concentrations ranged from 0.1 to 100 μM of curcumin in each well. Each well procedure was performed in triplicate (*n* = 6 per group).

The embryos were examined 24 (48 hpf) and 72 (96 hpf) hours after the addition of nanoparticles. Developmental disorders and delays and morphological changes, including irregular shape of the yolk sacs, impaired tail development, and decreased motor activity, were recorded.

To estimate the full range of mortality from 0 to 100%, embryonic deaths were recorded at 24 (48 hpf) and 72 (96 hpf) hours after addition of compounds. Toxicity assay (LC_50_ calculation) was determined based on cumulative mortality at the end of the experiment and was estimated using regression analysis.

### 2.5. Statistical Analysis

In cytotoxicity studies, the obtained data represented a normal distribution. The experiments were performed for no less than three times in three replicates. Statistical analysis was conducted using ordinary one-way ANOVA followed by Dunnett’s multiple comparisons test using Graphpad Prism 6.01 software (San Diego, CA, USA).

## 3. Results

### 3.1. Physicochemical Characteristics of PVP-Cur NPS

The basic properties, size, shape and ζ-potential of the PVP-Cur NPs were determined after resuspension of the resulting samples in water. The water resuspendability test showed that freeze-dried PVP-Cur NPs samples were easily dispersed back into distilled water and appeared translucent, similar to original nanoparticle dispersion before freeze-drying, which was confirmed by the results of the dynamic light scattering (DLS) analysis. All basic properties before and after freeze-drying are shown in Table 1.

Given that the loading of curcumin was about 10 wt.% of the polymer mass, the degree of encapsulation of biologically active substances in nanoparticles reached 95%.

**Figure 1:**
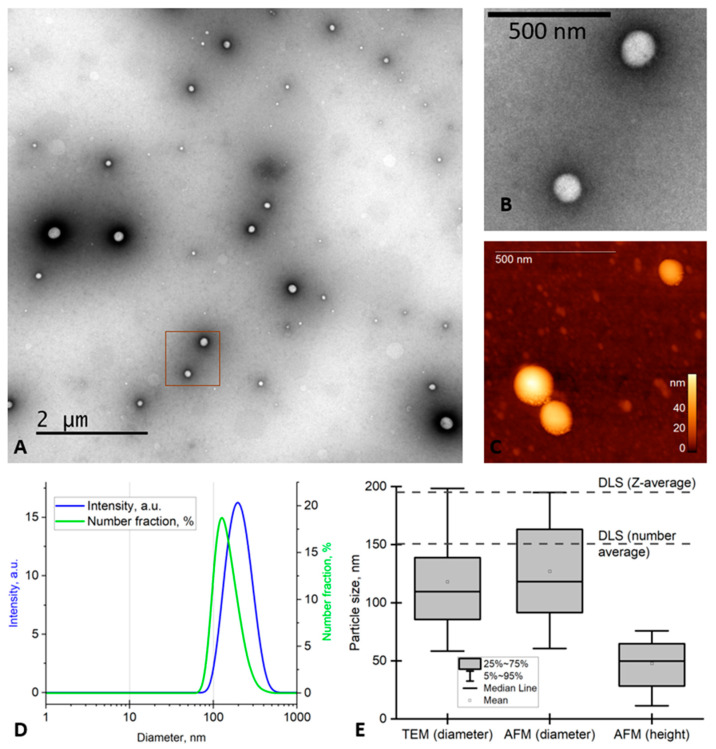
Characterization of the PVP-Cur NPs using TEM (**A**,**B**), AFM (**C**) and DLS (**D**) and the measured sizes (**E**). The image B shows the magnified part of image A. The dotted lines in E show the z-average and the number average obtained using DLS.

The PVP-Cur NPs were spherical particles, as shown by TEM (Figure 1A,B) and AFM (Figure 1C). For the AFM imaging, NPs were deposited onto TEM grids (amorphous carbon) rather than mica or graphite, which are commonly used as substrates. We chose the grids treated with the glow discharge because the adsorption of the NPs onto their surface was relatively high, and they were previously used as substrates for the AFM imaging [51].

The size of the NPs was measured using TEM, AFM and DLS, and the obtained data are summarized in Figure 1E.

The z-average size and the number average measured using DLS (190 nm and 150 nm, respectively (Figure 1D,E)) were higher than the mean particle size determined using microscopy. This is the difference between the particle hydrodynamic diameter measured by DLS and the particle projection diameter assessed by the AFM and TEM. A similar difference was observed for the poly(styrene-co-acrylic acid) copolymer particles [52]. The mean polydispersity index (*PDI*) of the NPs was 0.10 ± 0.03 by DLS, which confirms the homogeneity of the analyzed NPs. The *PDI* based on the TEM data can be easily calculated by the following formula:

$$PDI=({\frac{SD}{mean\;diametr})}^{2}$$

where *SD* is the standard deviation.

In our case, according to the TEM data, *SD* = 40 nm and mean diameter = 118 nm, so we obtained an estimate for *PDI* of ~0.11, which is in agreement with the value obtained using DLS.

Overall, the data obtained using the single-particle measurements (AFM, TEM) and ensemble measurements (DLS) were in good agreement.

### 3.2. Curcumin In Vitro Release Study

**Figure 2:**
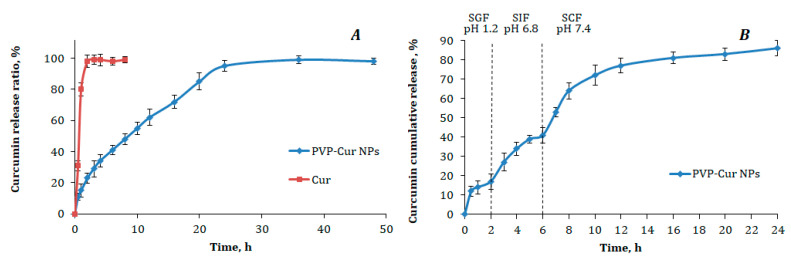
(**A**) Curcumin in vitro release profiles from PVP-Cur NPs and curcumin solution (Cur) in PBS (pH 7.4) at 37 °C; (**B**) cumulative curcumin release from the PVP-Cur NPs into simulated gastric fluids modeling three digestive system tracts. Data are plotted as the average ± SD of three measurements.

PVP-Cur NPs were incubated at 37 °C in PBS; the in vitro release profile studied over 48 h is presented as a cumulative release percentage in Figure 2A.

As one can see, a typical two-phase release profile was observed for the tested formulation when it was placed to the released medium. First, there was a relatively rapid release of about 10% of the loaded curcumin in the initial 30 min, followed by sustained release of the remaining curcumin by 24 h. In comparison, over 30% of the curcumin from the control water/methanol mixture was released into the medium rapidly by 30 min, with further almost complete curcumin recovery after 3 h.

The in vitro curcumin release study has been designed to simulate the human digestion tracks. The resulting curve of cumulative curcumin release from PVP-Cur NPs is presented in Figure 2B.

In the first medium, which simulated gastric fluid (pH 1.2) similarly to the abovementioned experiment, there was observed a slight burst effect, which can be explained mostly by the release of curcumin bound to the PVP outer shell. With the increase of the pH value of the media in simulated intestinal fluid (pH 6.8) and simulated colonic fluid (pH 7.4), higher curcumin amounts were released in a sustainable manner. The steps after 2 h and 6 h of the experiment were caused by the switch of the release media. The cumulative drug release for 24 h from the PVP-Cur NPs was about 85%. It can also be mentioned that only a rather small amount of curcumin was released into the fluid modeling the stomach’s acidic environment, while the highest amount of curcumin was released into the simulated colonic fluid.

During the stability test, no changes were observed in the content of curcumin in PVP-Cur NPs or in their average size in the accelerated stability studies (Table 2), which is evidence of rather good stability of the obtained PVP-Cur NPs.

### 3.3. Cytotoxicity Assay

**Figure 3:**
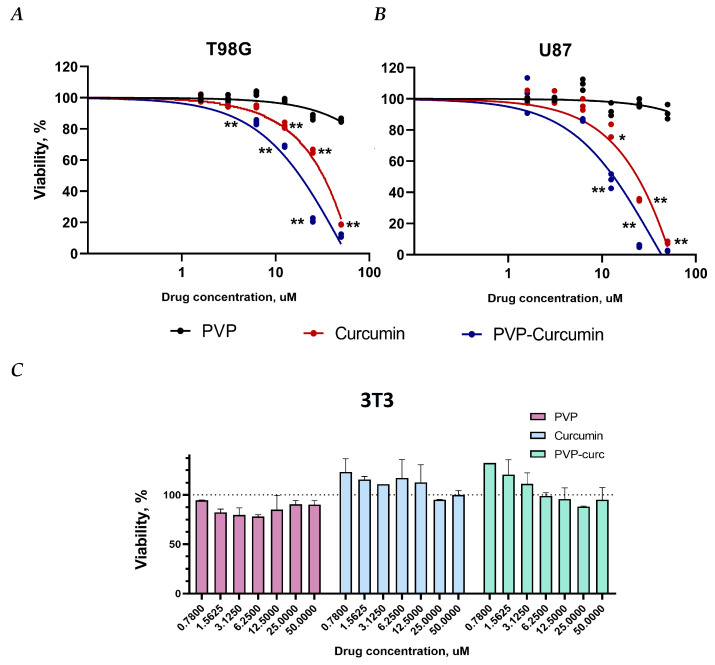
Cytotoxicity of PVP-Cur NPs, curcumin and PVP in (**A**) T98G, (**B**) U87 and (**C**) 3T3 cells. The data were analyzed using Graphpad Prism 6.01 software. Statistical analysis was performed using ordinary one-way ANOVA followed by Dunnett’s multiple comparisons test using Graphpad Prism 6.01 software (San Diego, CA, USA). * *p* < 0.05 and ** *p* < 0.005 indicate significant differences from the control.

The cytotoxicity of PVP-Cur NPs in comparison with curcumin and PVP was studied in glioblastoma cell lines T98G and U87 (Figure 3A,B). Normal NIH/3T3 mouse embryonic fibroblasts were used as control (Figure 3C). The viability of fibroblast cells exceeded 85% for free curcumin and PVP-Cur. Viability was above 85% for free PVP. The half-maximal inhibition concentration (IC_50_) of curcumin was 70.1 ± 3.5 μM for T98G cells and 48.6 ± 1.7 μM for U87 cells. The IC_50_ of PVP-Cur NPs was determined to be 29.3 ± 3.7 μM and 20.7 ± 1.3 μM for T98G and U87 cells, respectively.

### 3.4. Cell Uptake Assay

**Figure 4:**
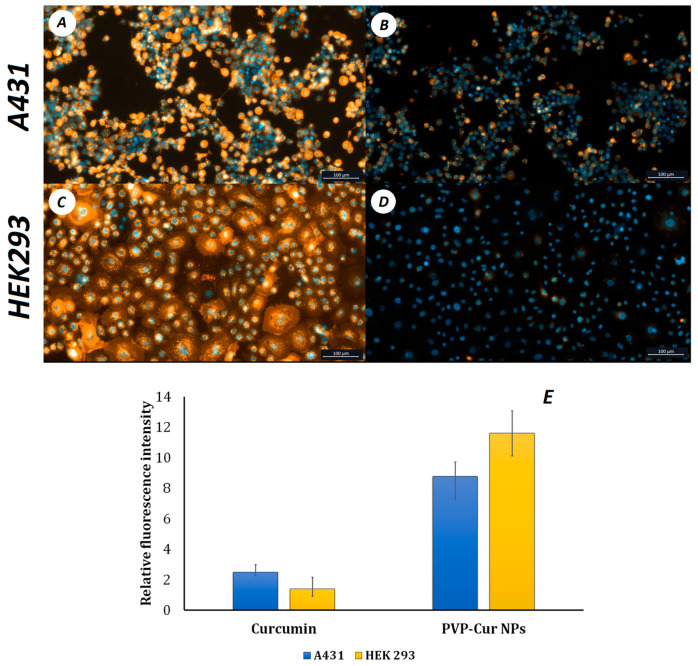
Uptake of PVP-Cur NPs (**A**,**C**) and free curcumin (**B**,**D**) by A431 and HEK 293 cells and relative fluorescence intensity after 60 min (**E**).

PVP-Cur NPs were used for in vitro uptake studies in A431 epidermoid carcinoma and HEK293 human embryonic kidney cells. Relative fluorescence intensities were compared 60 min after cells were treated with NPs and free curcumin. Sixty minute after treating cells with free curcumin and PVP-Cur NPs, it was shown that the relative fluorescence intensity of curcumin loaded into the nanoparticles was 1.5 times higher than that of the free curcumin (Figure 4). This difference in the absorption is due to the presence of an amphiphilic poly(N-vinylpyrrolidone) shell and correlates with other studies related to curcumin encapsulation [53,54].

### 3.5. Fish Embryo Acute Toxicity Test (FET)

**Figure 5:**
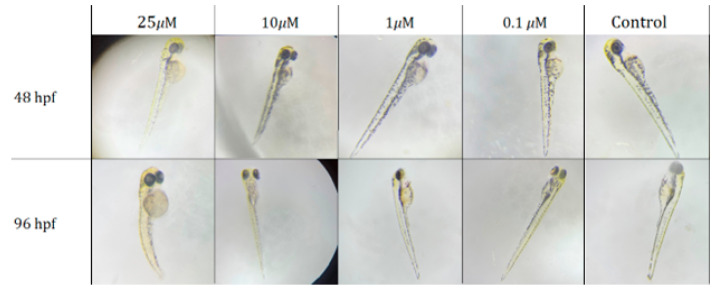
*Danio rerio* embryos aged 48 and 96 hpf after 24 h (48 hpf) and 72 h (96 hpf) of incubation with the studied curcumin nanoparticles at 40× magnification.

Zebrafish embryo treatment with PVP-Cur NPs in concentrations 25, 50 and 100 μM provided neurotoxicity that was characterized by increased motor activity of the tail (Figure 5).

**Figure 6:**
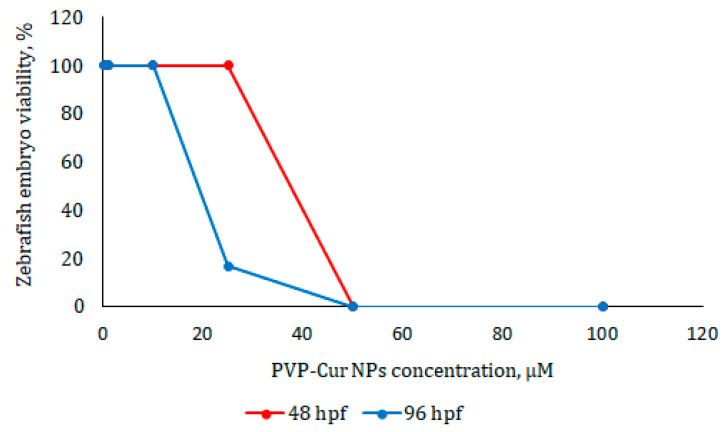
Survival of *Danio rerio* embryos (*n* = 6 per group) during 24 h (48 hpf) and 72 h (96 hpf) incubation with PVP-Cur NPs. Lethal concentration LC50 = 23.7 μM (per curcumin contents) was determined by regression analysis using Graphpad Prism version 6.01.

The death of all embryos was observed after 24 h of treatment with nanoparticles in concentrations of 50 and 100 μM per PVP-Cur NPs. Embryos that were treated with concentration of 25 μM had enlargement of the yolk sac and developmental delay after 24 h, which persisted 72 h after incubation, and 5 out of 6 embryos were found dead in 72 h of experiments. Concentrations of 10, 1 and 0.1 μM provided no toxic effects. The LC_50_ value was determined as 23.7 μM (Figure 6).

## 4. Discussion

We have previously shown the possibility of using amphiphilic PVPs with different molecular weights as drug carriers. In this work, we show that amphiphilic poly(N-vinylpyrrolidone) may be a suitable curcumin carrier for cancer treatment.

Amphiphilic poly(N-vinylpyrrolidone) was synthesized by radical polymerization. The molecular weight of the resulting polymer was 6 kDa. As it was shown previously, this molecular weight accounts for suitable critical aggregation concentration and low toxicity of the polymer [21,25,27]. PVP-Cur NPs were obtained by the solvent evaporation method: dispersion was carried out using an ultrasonic homogenizer using two infinitely compatible solvents (acetone/water). The samples obtained by this method have an average hydrodynamic diameter of about 200 nm as determined by DLS. It has been established that the ζ-potential ranges from −4 to −5 mV and differs little from the ζ-potential of the outer cell membrane, which also has a close negative value and can range from −2 to −90 mV depending on the cell type, the composition of a particular membrane region and the pH of the environment [55,56]. It should be noted that the electrical stabilization of low-molecular-weight surfactants is reflected by high absolute surface charge values, while for high-molecular-weight surfactants (i.e., amphiphilic polymers), stabilization is achieved through different mechanisms (i.e., steric stabilization) and is not clearly reflected by the absolute value of the surface charge [57].

The mean diameter of the particles was 118 ± 40 nm and 127 ± 41 nm (mean ± SD) according to TEM and AFM, respectively. The latter diameter was higher due to the AFM tip broadening. The height of the particles above the substrate (48 ± 40 nm) was far smaller than their lateral diameter, and the difference indicates intense flattening of the particles. Additionally, it can be a consequence of the variations in the tip–sample interaction, which shifts the cantilever resonance frequency [58]. A similar difference between the height and width measured using AFM was previously observed for viruses [59,60], polymer nanoparticles [23], DNA [61] and other samples.

As curcumin is a poorly water soluble and dispersible substance, its encapsulation strategy using polymeric micelle-like nanoparticles was studied and revealed as a successful way to improve this essential physicochemical property.

The prepared NPs showed favorable long-term stability. This improvement could be explained by the unique core–shell structure of polymeric micelle-like aggregates immobilizing curcumin as hydrophobic cargo in their hydrophobic inner core, while the PVP hydrophilic shell contributes to solubilizing of these nanoparticles in the aqueous media.

In current study PVP-Cur NPs showed an in vitro two-phase release profile in PBS (pH 7.4), composed of an immediate drug release followed by a sustained release. The two-phase delivery system helps in overcoming multiple dosing regimen problems. The initial burst release can be caused by the release of the drug entrapped in the polymeric shell of the nanoparticles, whereas the sustained release is the result of the drug diffusion from the hydrophobic core of the nanoparticles. Moreover, the outer shell PVP layer probably forms a diffusion obstacle for the released drug, resulting in a slower release. Therefore, obtained results imply that the release profile of the studied nano-scaled carrier systems is significantly affected both by hydrophobic interactions in the nanoparticle core and by the polymer coating layer.

The in vitro curcumin release investigation in simulated gastric fluids with a sequence of pH values of 1.2, 6.8 and 7.4 revealed that a low amount of curcumin release occurs at pH 1.2 and 6.8, while an increased extent of sustained release is discovered at pH 7.4.

The release profile shows sustained slow, gradual release of curcumin at each point of time from polymeric nanoparticles at a physiologic pH of 7.4. Sustained drug release from the nanoparticles is an important feature as it can dramatically increase the biologically active substance bioavailability and prolong diagnostic or therapeutic effect.

Curcumin obtains multiple effects in vivo, including antiproliferative, anti-inflammatory and antioxidant effects [62], which suggests that it can be utilized as a component of anticancer therapy. However, curcumin obtains poor stability in vitro and in vivo with a half-life of about 5 min, preventing it from exhibiting any therapeutically relevant effects [63]. Therefore, encapsulating curcumin into nanoparticles helps improve its bioavailability and ensures delivery to the lesion site, where it can exert its therapeutic properties.

In the current study, cytotoxicity assays showed that PVP-Cur NPs are non-toxic for healthy fibroblasts. Previously, the low cytotoxicity of PVP NPs, including those with curcumin, was shown for different cell types, including human embryonic stem cell derived fibroblasts (EBF-H9), human microvascular endothelial cells (HMEC-1), human embryonic kidney cells (HEK 293) and others [43,57,64,65,66].

The cell uptake assay showed that curcumin encapsulated in core/shell PVP-Cur NPs is transferred into the intracellular space 1.5 times more effectively than free curcumin. This difference in absorption is due to the presence of an amphiphilic poly(N-vinylpyrrolidone) shell, which is similar to liposome shells and correlates with other studies related to curcumin encapsulation. The mechanism of action is similar to the interaction of liposomes with cells. This is why the fluorescence of curcumin in cells is higher in the case of PVP-Cur NPs than in the case of free curcumin [67,68,69].

Due to accelerated cellular uptake of nanoparticles in vitro, the toxicity of PVP-Cur NPs increased compared to free curcumin against malignant cancer cells. This is consistent with the data previously obtained in [70]. Numerous studies demonstrate that curcumin can target signaling pathways involved in glioblastoma development: for example, by modulating the activity of transcription factors such as NF-κB and STAT3 and regulating the expression of genes implicated in malignant transformation and cell survival [71,72,73]. Curcumin is shown to modulate properties of glioblastoma stem cells via activation of autophagy [74]. Also, curcumin was reported to enhance the effect of radiation on glioma cells [75], making it a potential candidate for treatment of this type of cancer.

It can be assumed that such nanoparticles loaded with curcumin will be suitable for the treatment of malignant cancers, in particular glioblastoma. In vivo acute toxicity results on zebrafish indicated that below a concentration of 23.7 μM, PVP-Cur NPs are safe for use in living organisms.

## 5. Conclusions

Curcumin-loaded nanoparticles based on amphiphilic poly(N-vinylpyrrolidone) were synthesized in this study. The molecular weight of PVP was 6 kDa and was the most suitable due to its hydrophilic–hydrophobic balance. PVP-Cur NPs obtained by the solvent evaporation method had spherical form, an average hydrodynamic diameter of about 200 nm and the ζ-potential was between −4 and −5 mV. PVP-Cur NPs are non-toxic for healthy fibroblast cells and very toxic for glioblastoma cells. The IC_50_ of PVP-Cur NPs was determined to be 29.3 ± 3.7 μM and 20.7 ± 1.3 μM for T98G and U87 cells, respectively. In vivo experiments on zebrafish shows that PVP-Cur NPs do not have acute toxicity at concentrations below 23.7 μM. The in vitro release profiles in different media indicate that amphiphilic PVP nanoparticles have prolonged release action and can be considered as a prospective drug delivery system. Overall, the PVP-Cur NPs are promising for the treatment of malignant cancers without toxic effects for healthy cells and organs.

## Figures and Tables

**Table 1:** The main characteristics of the nanoparticles before and after freeze-drying.

PVP-Cur NPs	Z-Average Hydrodynamic Diameter (nm ± SD)	LC (% Mass ± SD)	LE (% Mass ± SD)	ζ-Potential (mV ± SD)
Before freeze-drying	191.1 ± 11.3	9.3 ± 0.3	93.9 ± 1.2	−4.00 ± 0.41
After freeze-drying	190.0 ± 12.2	9.2 ± 0.5	93.6 ± 1.1	−4.21 ± 0.15

**Table 2:** Accelerated stability studies of PVP-Cur NPs according to the ICH guidelines.

Time (Month)	Residual Curcumin Content (%)	Average Size (nm ± SD)
0	100.00	190.0 ± 12.3
1	99.04	191.2 ± 13.3
2	97.23	189.5 ± 12.8
4	96.62	190.4 ± 12.9
6	94.11	190.3 ± 13.7

## Data Availability

All data are publicly available via open access on journal website. Additional data are available upon request.
